# Accelerating targeted mosquito control efforts through mobile West Nile virus detection

**DOI:** 10.1186/s13071-024-06231-7

**Published:** 2024-03-18

**Authors:** Zsaklin Varga, Rubén Bueno-Marí, José Risueño Iranzo, Kornélia Kurucz, Gábor Endre Tóth, Brigitta Zana, Safia Zeghbib, Tamás Görföl, Ferenc Jakab, Gábor Kemenesi

**Affiliations:** 1 National Laboratory of Virology, Szentágothai Research Centre, University of Pécs, Pécs, Hungary; 2 Institute of Biology, Faculty of Sciences, University of Pécs, Pécs, Hungary; 3 Department of Research and Development, Laboratorios Lokímica, Valencia, Spain; 4 Parasite & Health Research Group, Department of Pharmacy, Pharmaceutical Technology and Parasitology, Faculty of Pharmacy, University of Valencia, Valencia, Spain

**Keywords:** Mosquito-borne pathogens, Surveillance, Prevention, Field-based, Rapid diagnostics, Bagaza, Panflavivirus, Heminested-PCR, NGS sequencing

## Abstract

**Background:**

Different mosquito control strategies have been implemented to mitigate or prevent mosquito-related public health situations. Modern mosquito control largely relies on multiple approaches, including targeted, specific treatments. Given this, it is becoming increasingly important to supplement these activities with rapid and mobile diagnostic capacities for mosquito-borne diseases. We aimed to create and test the applicability of a rapid diagnostic system for West Nile virus that can be used under field conditions.

**Methods:**

In this pilot study, various types of adult mosquito traps were applied within the regular mosquito monitoring activity framework for mosquito control. Then, the captured specimens were used for the detection of West Nile virus RNA under field conditions with a portable qRT-PCR approach within 3–4 h. Then, positive samples were subjected to confirmatory RT-PCR or NGS sequencing in the laboratory to obtain genome information of the virus. We implemented phylogenetic analysis to characterize circulating strains.

**Results:**

A total of 356 mosquito individuals representing 7 species were processed in 54 pools, each containing up to 20 individuals. These pools were tested for the presence of West Nile virus, and two pools tested positive, containing specimens from the *Culex pipiens* and *Anopheles atroparvus* mosquito species. As a result of subsequent sequencing, we present the complete genome of West Nile virus and Bagaza virus.

**Conclusions:**

The rapid identification of infected mosquitoes is the most important component of quick response adulticide or larvicide treatments to prevent human cases. The conceptual framework of real-time surveillance can be optimized for other pathogens and situations not only in relation to West Nile virus. We present an early warning system for mosquito-borne diseases and demonstrate its application to aid rapid-response mosquito control actions.

**Graphical Abstract:**

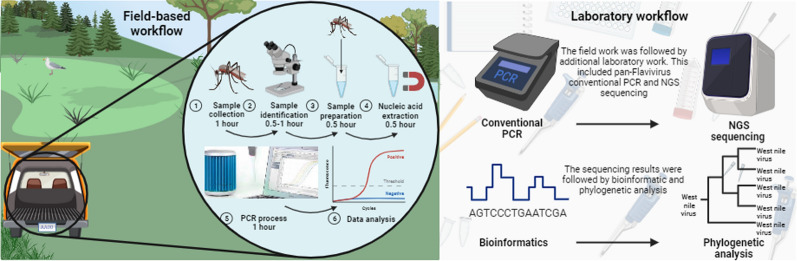

**Supplementary Information:**

The online version contains supplementary material available at 10.1186/s13071-024-06231-7.

## Background

There are multiple endemic mosquito-borne viruses in Europe with human or animal health relevance. In recent decades an increasing number of autochthonous cases were detected with these viruses [1, 2]. In addition, exotic viruses are also emerging in some parts of Europe, such as Dengue virus with local transmission in France in 2022 [3] and in Italy in 2023 [4].

One of the most significant endemic human pathogenic mosquito-borne viruses in Europe is the West Nile virus (WNV). WNV is a positive-sense RNA virus from the *Flaviviridae* family. The virus has a complex life cycle involving birds, mosquitoes, and humans. The virus is transmitted to humans through the bite of an infected mosquito. The mosquito gets the virus from biting an infected bird. The virus then replicates in the mosquito's body and is passed on to humans when the mosquito bites them. It can cause fever, headache, body aches, nausea, vomiting, and sometimes swollen lymph glands or a skin rash. In severe cases, West Nile virus can cause neurological illnesses such as encephalitis or meningitis [5–7].

It was first described in the continent in 1960; since then, the virus has appeared in many European countries such as France, Cyprus, Portugal, Hungary, etc. [8]. In these countries, genetic lineage 1 and lineage 2 of the virus have been responsible for human and animal infections so far [9].

In Spain, the most significant region of WNV circulation is the southern part of the country where multiple outbreaks were recorded based on the data from the last decade [2, 10–12]. The year 2020 was exceptional for WNV activity, when an outstanding WNV epidemic took place in southern Spain, affecting Andalusia, Seville, Catalonia, and Valencia, causing 77 human cases and 8 deaths [13–15].

It is most commonly found in Africa, the Middle East, and parts of Asia, but it has also been found in Europe, North America, and Australia.

West Nile virus has a complex life cycle involving birds, mosquitoes, and humans. The virus is transmitted to humans through the bite of an infected mosquito. The mosquito becomes infected when it feeds on an infected bird. The virus then replicates in the mosquito's body and is passed on to humans when the mosquito bites them.

*Culex pipiens* mosquito (Linnaeus, 1785) is considered as the primary vector of WNV but actual vector competence may vary between regions and other species can also contribute to its spread. Based on available literature data, some *Aedes* species, such as *Aedes albopictus* (Skuse, 1894), and members of the genus *Anopheles* (Meigen, 1818) can be considered competent vectors as well [16–21].

It is also a common attribute of Flaviviruses that multiple viruses are naturally co-circulating between avian and mosquito hosts in the same ecosystem. WNV often co-circulates with Bagaza or Usutu viruses [22]. Bagaza virus (BAGV) belongs to the Flaviviridae family, Ntaya serocomplex. It was first isolated from *Culex* mosquito species in the 1966 outbreak in Bagaza, Central Africa [23]. The virus is pathogenic in red-legged partridges and caused an outbreak in Cadiz, Spain, in 2019 [24].

There are multiple mosquito monitoring programmes in Europe, using different strategies, but most of the data still come from event-based (human cases) and indicator-based surveillance activities [25–27]. Whether it is mosquito surveillance or event-based data, the resulting data will not be immediately available; they will take time to be processed in the laboratory or made available in the ECDC (European Centre for Disease Prevention and Control) [25–27]. Thus, the necessary actions are not real time. In this study we demonstrate a WNV detection approach that uses on-site PCR technique to provide rapid surveillance results in 3–4 h, which is a rapid protocol that can be implemented in the field, followed immediately by the necessary targeted control after virus detection. We complemented the surveillance activity with additional complete genome sequencing in the laboratory and provided genome data of BAGV and WNV from southern Spain in 2021.

## Methods

### Trapping and virus detection

**Fig. 1:**
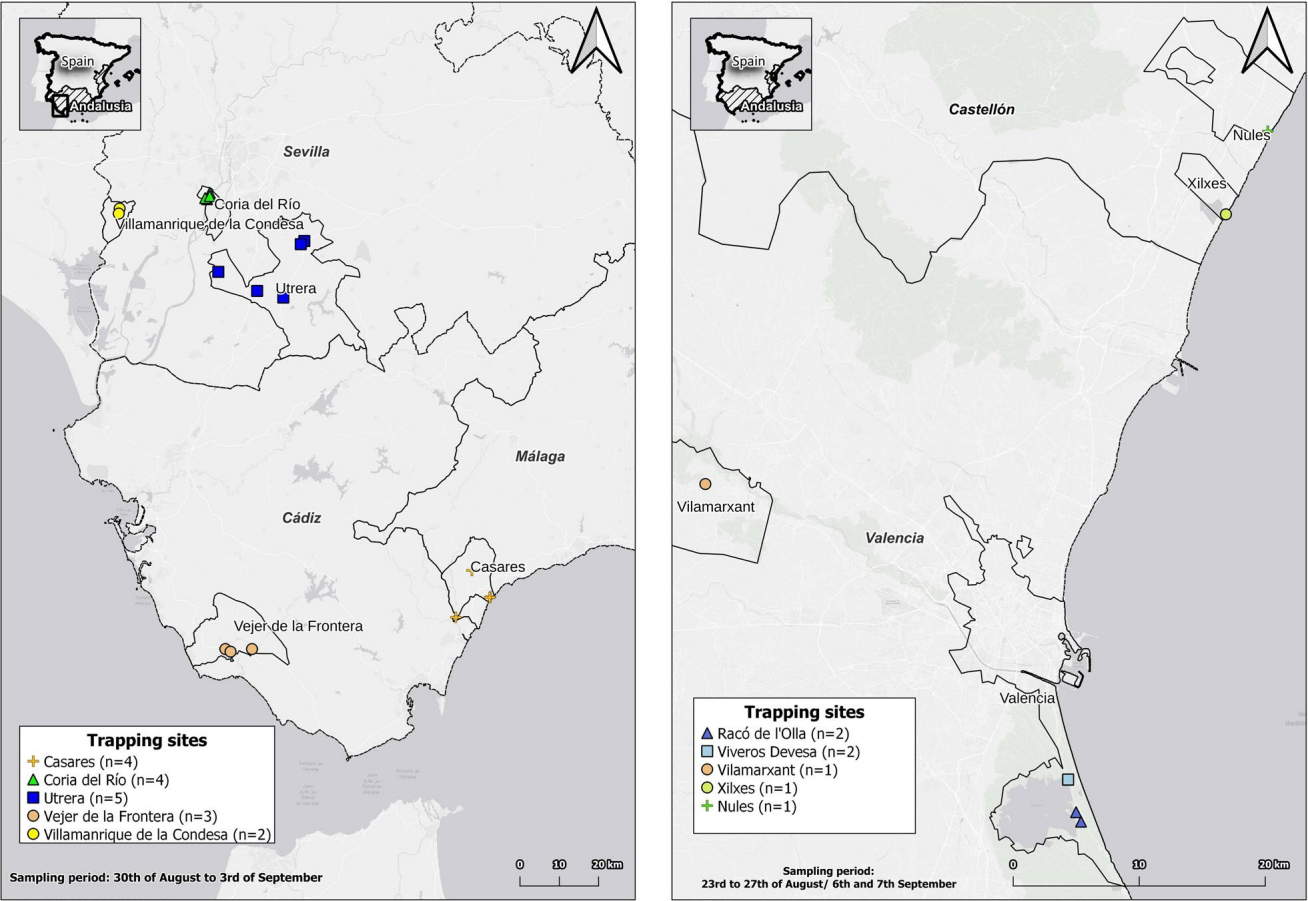
Mosquito sampling sites in the provinces of Valencia (7) and Andalusia (18), Spain

Adult mosquito collection was carried out in two Spanish regions: Valencia and Andalusia, during the WNV season, between 23 August and 8 September 2021 with overnight trapping. During overnight trapping (from 6 p.m. to 8 a.m.), trap nets were changed daily, except in a few cases because of the distance between sites and weather conditions. If a trap did not catch any or caught insufficient quantities on a given night, the net was left out for a longer period. Details of each sample location are summarized in Additional file 1: Table S1. Based on data from previous years, 25 sites were selected, mostly in suburban areas, with abundant local mosquito populations [28]. Trapping took place at 7 sites in the province of Valencia and 18 sites in Andalusia (Fig. 1). Various sampling methods were used mostly with BG-Sentinel traps (both with lure and CO_2_) and in some cases supplemented with CDC Light traps (with yeast as CO_2_ source) or BG-Mosquitaire (with lure). As the type of trap was not important in the testing of our protocol, any other type of adult mosquito trap can be used for the work. After collection from traps, a freezing box was used as an euthanization tool for the mosquitoes. The box operated with general ice accumulators, which were pre-frozen in −20 °C freezers. Following the freezing cycle, the mosquitoes underwent sex separation and morphological identification [29]. Sampling details are summarized in Table 1.

**Table 1:** Pools processed and tested for West Nile virus

Place (e.g. town, village)	Trap type	Mosquito species	Number of females	Pool ID	Positivity/Ct value
Valencia, Viveros Devesa	BG sentinel + BG lure7	*Aedes albopictus*	4	1	Negative
Valencia, Racó de l'Olla	BG sentinel + BG lure 7	*Culex pipiens*	1	2	Negative
Valencia, Viveros Devesa	BG sentinel + BG lure 7	*Aedes albopictus*	1	3	Negative
Valencia, Racó de l'Olla	BG sentinel + BG lure 7	*Aedes albopictus*	4	4	Negative
Valencia, Viveros Devesa	BG sentinel + BG lure 7 + CO2	*Aedes albopictus*	8	5	Negative
Valencia, Racó de l'Olla	BG sentinel + BG lure 7 + CO2	*Aedes albopictus*	3	6	Negative
Valencia, Villamarxant	CDC-light trap	*Culex pipiens*	3	7	Negative
Andalusia, Coria	BG sentinel lure	*Anopheles atroparvus* *Culex pipiens*	3 2	8/1 8/2	Positive / 17 Positive / 28
Andalusia, Utrera	BG sentinel lure	*Culex pipiens*	50	9/1	Negative
Andalusia, Utrera	BG sentinel lure	*Culex pipiens*	16	10	Negative
Andalusia, Coria	BG sentinel lure	*Culex perexiguus* *Culex pipiens*	1 2	12/1 12/2	Negative Negative
Andalusia, Coria	BG sentinel lure	*Culex pipiens*	1	13/1	Negative
Andalusia, Utrera	BG sentinel lure	*Culex pipiens*	1	14	Negative
Andalusia, Utrera	BG sentinel lure	*Culex pipiens*	3	15	Negative
Andalusia, Utrera	BG sentinel lure	*Anopheles atroparvus* *Culex pipiens*	3 1	16/1 16/2	Negative negative
Andalusia, Utrera	BG sentinel lure	*Culiseta longiareolata* *Culex pipiens*	2 19	17/1 17/2	Negative Negative
Andalusia, Coria	BG sentinel lure	*Culex pipiens*	2	18	Negative
Andalusia, Utrera	BG sentinel lure	*Culex pipiens*	1	19	Negative
Andalusia, Villamanrique	BG sentinel lure	*Culex pipiens*	10	20	Negative
Andalusia, Casares Sierra	BG sentinel lure	*Culex pipiens* *Aedes albopictus* *Culiseta longiareolata* Culex theileri	4 14 1 1	21/1 21/2 21/3 21/4	Negative Negative Negative Negative
Andalusia, Vejer de la Frontera	BG sentinel lure	*Culex pipiens*	12	22	Negative
Andalusia, Vejer de la Frontera	BG sentinel lure	*Culex pipiens* *Culex perexiguus*	3 2	23/1 23/2	Negative Negative
Andalusia, Coria	BG sentinel lure	*Anopheles atroparvus* *Culex perexiguus*	3 1	24/1 24/2	Negative Negative
Andalusia, Utrera	BG sentinel lure	*Culex pipiens*	1	25	Negative
Andalusia, Utrera	BG sentinel lure	*Culex pipiens*	2	26	Negative
Andalusia, Coria	BG sentinel lure	*Culex perexiguus* *Culex pipiens* *Anopheles atroparvus*	2 2 8	27/1 27/2 27/3	Negative Negative Negative
Andalusia, Casares—Secadero	BG sentinel lure	*Aedes albopictus* *Culex pipiens*	1 1	28/1 28/2	Negative Negative
Andalusia, Los Palacios y Villafranca (Utrera)	CDC-light trap	*Culex perexiguus* *Culex pipiens*	1 1	29/1 29/2	Negative Negative
Andalusia, Villamanrique	BG sentinel lure	*Culex pipiens*	2	30	Negative
Andalusia, Coria	BG sentinel lure	*Anopheles atroparvus* *Culex pipiens* *Culex perexiguus*	2 2 1	31/1 31/2 31/3	Negative Negative Negative
Andalusia, Vejer de la Frontera	BG sentinel lure	*Culex pipiens* *Culex perexiguus*	49 5	32/1 32/2	Negative Negative
Valencia, Nules	BG-mosquitaire	*Culex pipiens* *Anopheles atroparvus*	1 5	33/1 33/2	Negative Negative
Valencia, Xilxes	BG-mosquitaire	*Anopheles atroparvus*	8	34	Negative
Valencia, Viveros Devesa	Lab (larvae dipping)	*Aedes albopictus*	4	35	Negative
Valencia, Racó de l'Olla	Lab (larvae dipping)	*Culiseta longiareolata*	1	36	Negative
Valencia, Nules	BG-mosquitaire	*Aedes caspius* *Aedes detritus*	32 30	37/1 37/2	Negative Negative

Female mosquitoes, due to their blood-feeding behaviour, play an active role in virus transmission. Analysing these mosquitoes for the presence of the virus provides direct evidence of virus circulation in the region. Therefore, we focused on processing female mosquitoes, systematically categorizing and grouping them by species, sampling site, and collection date. This approach was employed to facilitate targeted West Nile virus (WNV) testing in separate, well-defined pools. To align with our sampling numbers and ensure rapid and efficient sample processing, we decided to use 20 individuals per pool.

Our concept aimed to streamline the detection process using mobile devices and minimize the associated laboratory procedures, so we selected the following methods and types of equipment accordingly. Samples were homogenized manually (sterile quartz sand and 500 µl PBS buffer were added to each pool) using sterile single-use plastic sticks. Total RNA was extracted using the Beckman Coulter RNAdvance Viral XP 1.5-ml Tube Protocol (Beckman Coulter, Inc., CA, USA), following the manufacturer’s protocol. For the magnetic bead-based nucleic acid extraction we used the MagJET Separation Rack (Thermo Fisher Scientific^™^). Samples were tested for WNV RNA by qRT-PCR with previously published primers [30]. Primers WN10533-10552 (AAG TTG AGT AGA CGG TGC TG) and WN10625-10606 (AGA CGG TTC TGA GGG CTT AC) were used to amplify a conserved 92-bp region of the WNV 3—noncoding region. Besides probe WN10560-10579 (CTC AAC CCC AGG AGG ACT GG) was used for the qPCR. Briefly, the qRT-PCR was performed on MyGo^®^ Mini S Real-Time PCR instrument (IT-IS Life Science Ltd.), a compact PCR machine that is suitable for use in the field, using the Brilliant III Ultra-Fast QPCR Master Mix (Agilent Technologies, CA, USA).

The total qRT-PCR master mix was 15 µl containing a total of 1 µl primers (50 µM), 0.25 µl probe (50 µM), 10 µl Mastermix, 0.2 µl dithiothreitol, 1 µl RT block enzyme, and 2.55 µl nuclease-free water. The PCR master mixes used in the field were prepared in advance and delivered frozen to the site; then, 5 µl extracted RNA of the samples (templates) was added to the master mix on site. The thermal cycling programme was set as follows: 10 min at 50 ℃ for reverse transcription, 3 min at 95 ℃ for denaturation, and 45 cycles of 10 s at 95 ℃ and 25 s at 60 ℃ for amplification [30]. All steps are field compatible, extraction does not require a homogenizer or centrifugal step because of the beads, and the PCR machine is compact and portable. Thanks to these features, the full protocol for in situ WNV detection from emptying the traps to PCR results takes about 3–4 h with the above-mentioned conditions, depending on the number of investigated mosquitoes.

To confirm the results of the above-mentioned in situ WNV detecting protocol, following the field-based surveillance activity, a previously published heminested RT-PCR was performed on the positive pools with 5 µl sample under laboratory conditions [31], and the amplified 250 bp of the NS5 gene was sent for Sanger sequencing (Eurofins Genomics Sequencing Laboratory, Germany). The results of the Sanger sequencing were inconclusive, showing mixed amplicons. Therefore, we decided to use Illumina sequencing on the *Anopheles atroparvus* pool to obtain the whole genomes and to distinguish possible mixed sequences.

### Genome sequencing and analysis

Following the total nucleic acid isolation and reverse transcription, DNA was amplified by random PCR. RNA library was generated using the NEBNext Ultra II Directional RNA Library Prep for Illumina (NEB, Ipswitch, MA, USA). Briefly, 10 ng of total RNA was used as input for fragmentation step, and the cDNA generation was performed using random primers. Thereafter, the cDNA was end-prepped and adapter-ligated; then, the library was amplified according to the manufacturer’s instructions. The quality of the libraries was checked on Agilent 4200 TapeSation System using D1000 Screen Tape (Agilent Technologies, Palo Alto, CA, USA); the quantity was measured on Qubit 3.0 (Thermo Fisher Scientific, Waltham, MA, USA). Illumina sequencing was performed on the NovaSeq 6000 instrument (Illumina, San Diego, CA, USA) with 2 × 151 run configuration. Raw reads were quality checked with FastQC v0.12.1 and error corrected and quality trimmed with NanoFilt v2.8.0. Genomes and genome parts were de novo assembled with SPAdes v3.15.5 (raw reads as SPAdes has a built-in error correction and quality trimming function) and MEGAHIT v1.2.9 (corrected reads) and were mapped to the closest matches in Genbank in Geneious Prime v2023.1.1. Illumina reads were mapped to the consensus sequences from the former step and further corrected in Geneious Prime v2023.1.1. For multiple sequence alignments, sequence, and phylogenetic analyses, Geneious Prime 2023.1.1 and PhyML software version 3.0 were used. Phylogenetic analysis of WNV sequence was performed using PhyML software version 3.0. Model selection was accomplished using the model selection algorithm built into the software [32]. The model selection was run according to the Bayesian information criterion [33]. The best model was the GTR + G + I with 1000 bootstraps. For BAGV neighbour-joining phylogenetic tree was inferred with 1000 bootstrap replicates in Geneious Prime 2023.1.1.

## Results and discussion

An increasing body of research is endorsing the transition from event-based surveillance to forecasting or early warning system approaches in surveillance practices [34, 35]. Most of the studies are based on virus surveillance by detecting WNV from human samples or by processing mosquitoes that have been collected over several years [36–39].

During the field sampling, 356 adult mosquitoes were trapped representing 7 species. Occasionally, where larvae were present on site, we collected them with larva dipping method, although only two pools were included in the study with this method (Additional file 1: Table S1). Among the 54 pools that were processed for WNV testing on site by qRT-PCR, two pools were positive for WNV RNA. The two positive pools contained two individuals of *Culex pipiens* (28 Ct) and three individuals of *Anopheles atroparvus* mosquitoes (17 Ct) from the village of Coria, Andalusia, respectively. This is in line with evidence showing that Andalusia became a WNV hotspot during the last few years [39]. During the identification process, we observed that the abdomens of the mosquitoes were empty. This observation led us to conclude that the positivity for the West Nile virus originated from the mosquitoes themselves rather than from a blood meal they may have ingested.

Only partial genomic data were recovered during the confirmatory PCR experiments from the pool containing *Cx. pipiens*, probably because of low virus titres. Therefore, this sample was not subjected to NGS sequencing. However, we were able to retrieve complete and nearly complete viral genomic sequences from the *An. atroparvus* sample. The mean coverage was 127.01X ± 594.42 and 111.49X ± 538.62 in case of WNV and Bagaza genomes, respectively. Illumina sequencing verified the mixed positive status of the sample containing West Nile and Bagaza viruses (Genbank accession numbers OR472391 and OR472392). Based on these results we documented the co-circulation of these viruses in the region within *An. atroparvus* species. Although members of the genus *Anopheles* are not considered as primary vectors for WNV, multiple literature data present positive specimens from Europe; therefore, their vector role cannot be ruled out [16, 18–20, 40]. In Portugal, the role of *Anopheles* mosquitoes in the spread of the virus was described in the 1970s and the ECDC also lists members of the genus as potential vectors of WNV [22, 41–46]. The fact that we detected the virus in this species does not prove beyond doubt that it is a vector of the virus, but it is important to draw attention to the possibility and the role of the species in the virus circulation.

**Fig. 2:**
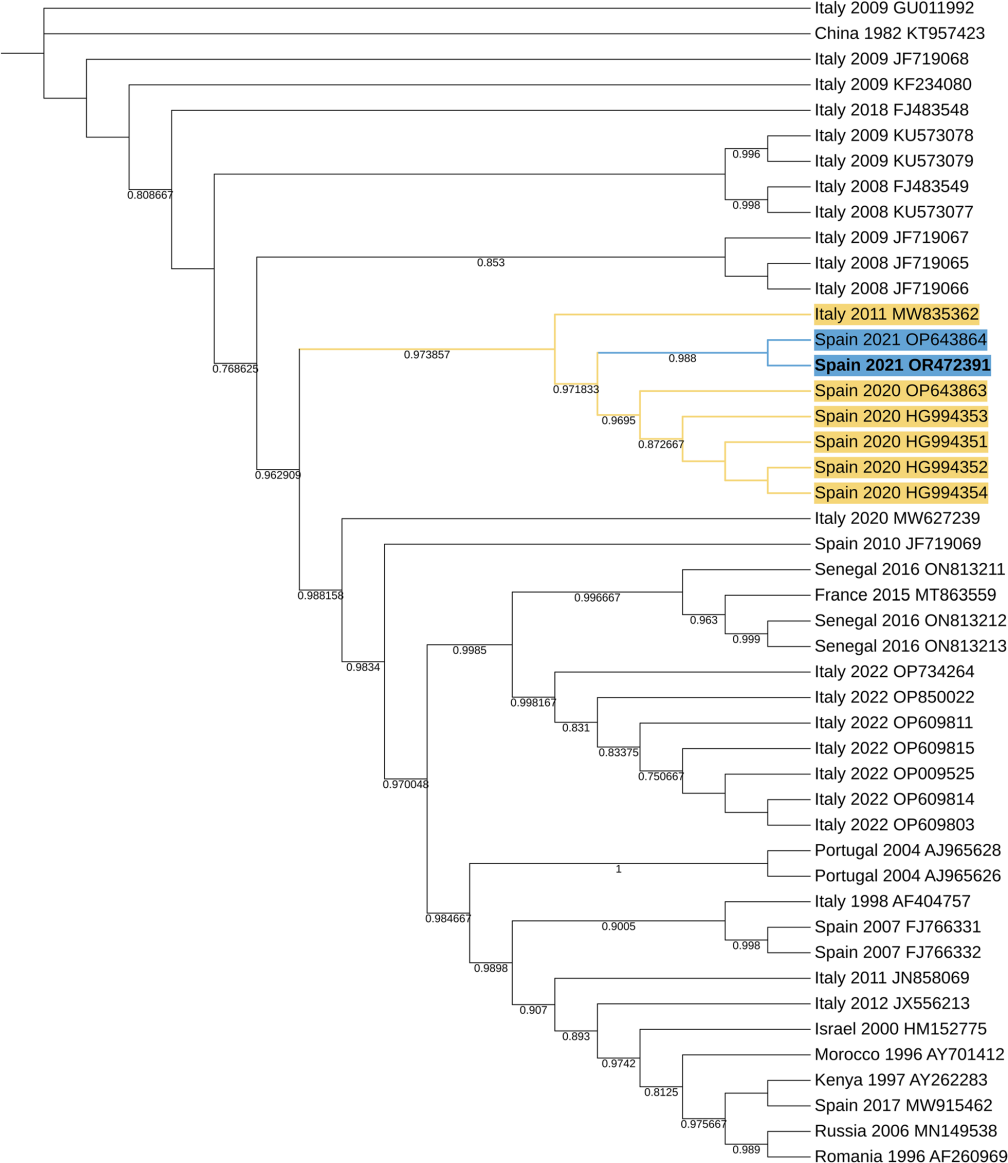
PhyML tree of West Nile virus sequences with 1000 bootstrap replicates as the test of phylogeny. The novel sequence data of this study are presented in bold letters. Yellow and blue colours are used for visual clarity. They highlight the node tip labels on the phylogenetic tree that are closest to the sequences we have described

**Fig. 3:**
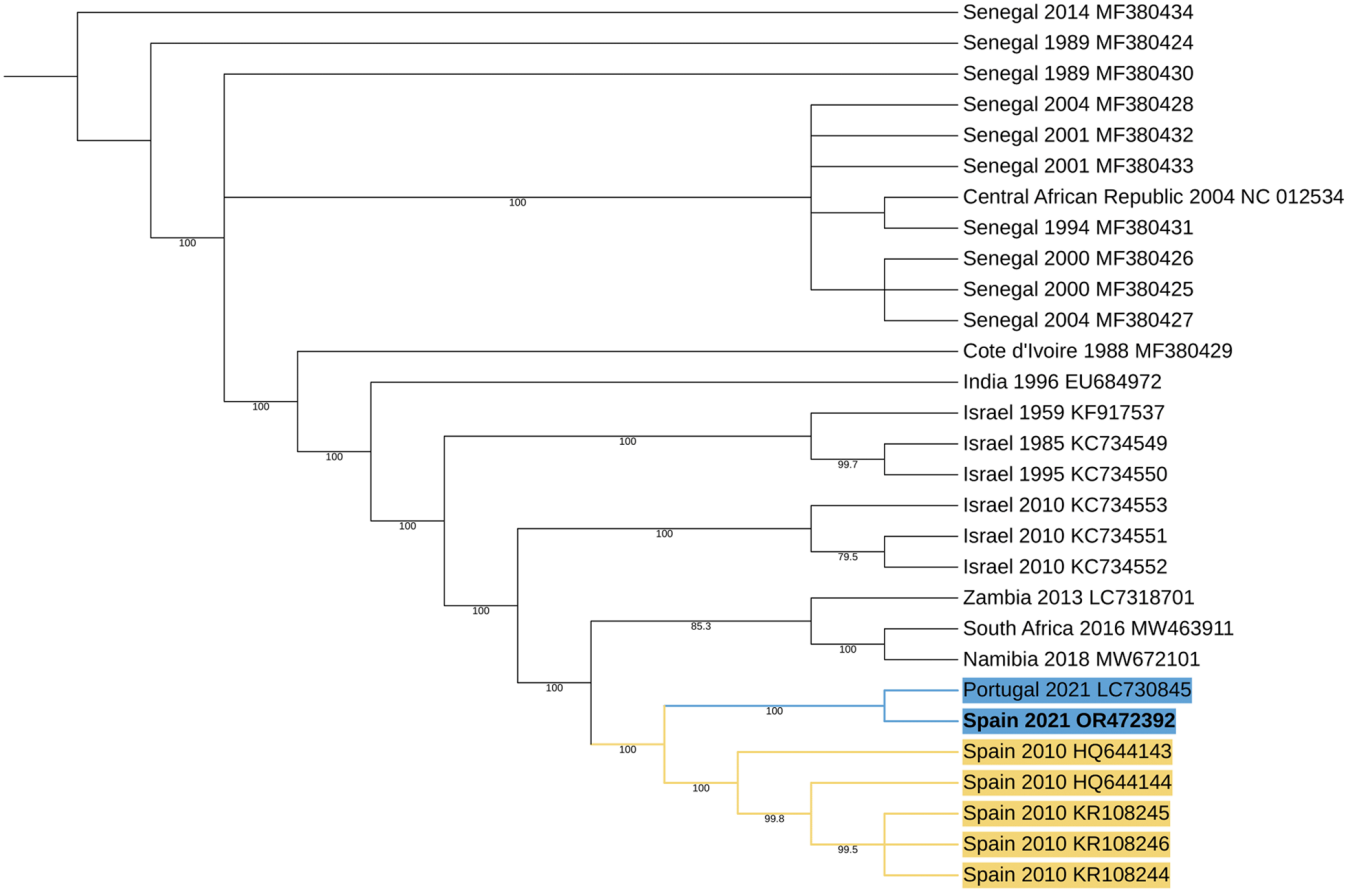
Neighbour-joining tree of Bagaza virus sequences. Neighbour-joining phylogenetic tree was inferred, using 1000 bootstrap replicates in Geneious Prime^®^ 2023.1.1 Novel sequence data of this study are presented in bold letters. Yellow and blue colours are used for visual clarity. They highlight the node tip labels on the phylogenetic tree that are closest to the sequences we have described

Using the complete genome sequences of BAGV and WNV, we performed phylogenetic analyses (Figs. 2, 3).

Our results indicate that the WNV sequence belongs to Lineage 1 and clusters together with sequences from *Culex perexiguus* collected in 2020 and 2021 in Spain and human samples collected in 2020 in Spain. This novel sequence belongs to the same phylogenetic cluster, the WNV lineage 1, clade 1a, Mediterranean subtype. Among these, the WNV sequence of this study is more closely related to the *Cx. perexiguus* sequence from 2021 [39]. Similarly to our positive sample, sequences from both earlier *Cx. perexiguus* mosquitoes and the human samples originate from near Coria, one of the centres of the 2020 human outbreak. In addition, it is important to note that our sequences (both WNV and BAGV) were found in *An. atroparvus*, highlighting the potential of other mosquito species as vectors or players in sustaining the endemic transmission of the virus. Also, it highlights the importance of expanding the target species for future surveillance studies beyond *Culex* species.

The phylogenetic position of the newly described BAGV sequence aligns with that of a Portuguese red-legged partridge sequence from 2021, indicating its membership in the G4 group of the Ntaya serogroup, within the BAGV/ITV monophyletic cluster [2, 47, 48] (Fig. 3).

Co-circulation of different Flaviviruses in the same ecosystem was reported in multiple localities across Europe, usually involving Usutu, West Nile, and Bagaza viruses [11, 49, 50].

BAGV is a zoonosis which is increasingly gaining attention as a potential and more significant veterinary pathogen in Europe with increasing frequency of detections. The 2019 outbreak of BAGV in Spain occurred in the area where WNV, USUV, and BAGV were confirmed to co-circulate in 2011. Red-legged partridges found dead during the outbreak had enlarged livers and kidneys and other poor body condition. This is a cause for concern, among other things because of its role in the ecosystem. In addition to animal health relevance, during the Indian outbreak, anti-BAGV antibodies were detected in humans, raising awareness that humans may also be exposed to the virus at some level [11, 51].

## Conclusions

In the present paper, we demonstrated the feasibility of our on-site surveillance line of action in mosquito-borne pathogen monitoring. It may be a valuable approach to aid rapid-response mosquito control actions and outbreak investigation activities or a good alternative for outbreak early warning systems, specifically in low-resource regions where mobile solutions can overcome logistic challenges. In addition to demonstrating this approach, we have released new genome sequences for both Bagaza and West Nile viruses from Europe. Moreover, we have identified another potential mosquito vector, *An. atroparvus*, in the region, thereby reinforcing the practicality and viability of these methods. To the best of our knowledge, this is the first in situ surveillance study for WNV.

### Supplementary Information

**Additional file 1: Table S1.** Mosquito trapping data. **Table S2.** NGS sequencing genome coverage plot and raw data for West Nile virus. **Table S3.** NGS sequencing genome coverage plot and raw data for Bagaza virus.

## Data Availability

All data presented in this paper are available in the additional files. All of the DNA sequences are uploaded to the GenBank and accession numbers are publicly available (OR472391, OR472392).
